# Stents versus bypass surgery: 3-year mortality risk of patients with coronary interventions aged 50+ in Germany

**DOI:** 10.1186/s13019-022-02014-2

**Published:** 2022-10-01

**Authors:** Sophia Nestler, Daniel Kreft, Peter Donndorf, Hüseyin Ince, Gabriele Doblhammer

**Affiliations:** 1 Institute of Sociology and Demography, University of Rostock, Ulmenstraße 69, 18057 Rostock, Germany; 2 German Center for Neurodegenerative Diseases (DZNE), Venusberg-Campus 1/99, 53127 Bonn, Germany; 3 Department for Cardiac and Vascular Surgery, Hospital of Karlsburg, Greifswalder Str. 11, 17495 Karlsburg, Germany; 4 Department of Cardiology, Rostock University Medical Center, Ernst-Heydemann-Straße 6, 18057 Rostock, Germany

**Keywords:** Coronary artery disease, Stents, Coronary artery bypass, Health claims data, Follow-up, Mortality

## Abstract

**Objectives:**

Due to demographic aging, the prevalence of coronary artery disease (CAD) is expected to increase in the future, resulting in a growing demand for stent and bypass interventions. This study aims to investigate the mortality risk of patients following conventional coronary artery bypass grafting (CABG) or endovascular procedure by the implantation of bare-metal stents (BMS) or drug-eluting stents (DES).

**Methods:**

Based on a random sample of 250,000 members of Germany’s largest health insurance ‘Allgemeine Ortskrankenkassen’ (AOK) from 2004 to 2015, incident CAD patients were analyzed by Cox Proportional-Hazard models. Risk adjustment was made for sex, age, other cardiac diseases, non-cardiovascular comorbidities and years since intervention. Due to later admission of DES and thus a shorter observation time, mortality was examined for 3 years since the intervention.

**Results:**

BMS represented the most frequent procedure (48%). We found similar proportions of CABG (19%) and DES interventions (23%). After risk adjustment, the models showed a 21% (*p* = 0.004) lower mortality risk of patients with DES and also a 21% (*p* = 0.002) lower mortality risk of CABG patients compared to persons with BMS.

**Conclusion:**

Based on a large-scale dataset, our study demonstrated survival advantages of CABG and DES interventions over BMS, with no differences between the DES and CABG groups. The results help to assess the risks of coronary interventions. Aspects of quality of life, severity of postoperative physical limitations, duration of rehabilitation, patients’ preferences, and aspects of cost-effectiveness for hospitals and society should be further considered.

**Graphical abstract:**

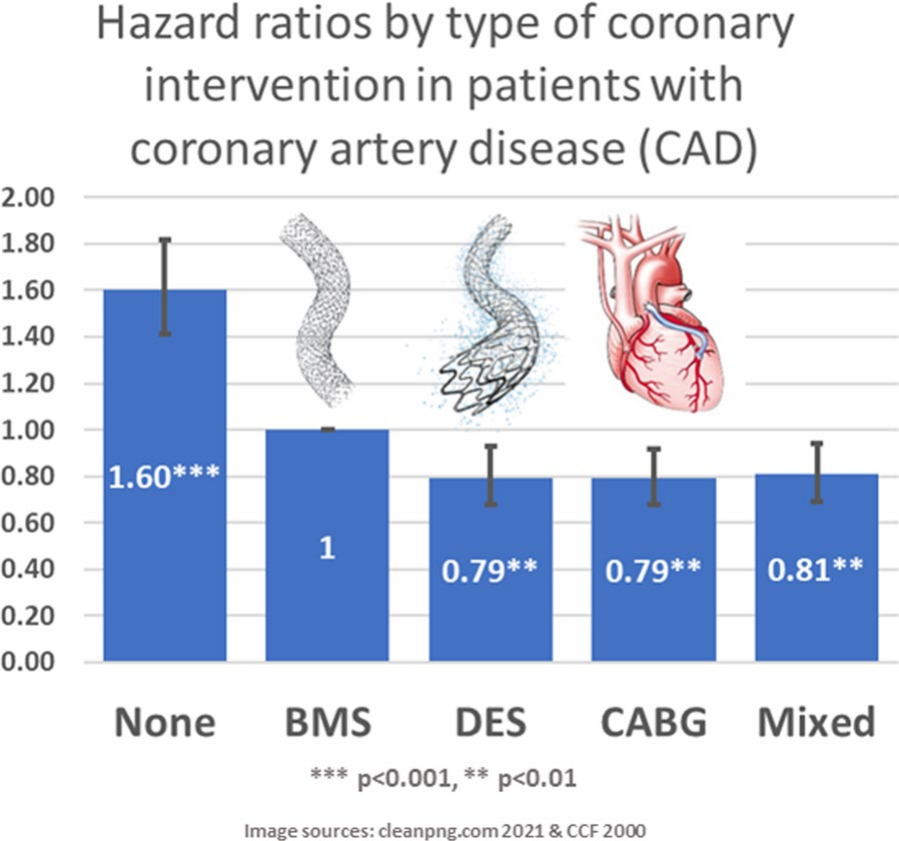

**Supplementary Information:**

The online version contains supplementary material available at 10.1186/s13019-022-02014-2.

## Introduction

Coronary artery disease (CAD) is the leading cause of death worldwide. Globally, ischemic heart diseases caused 8.9 out of 55.5 million deaths between 1980 and 2015 (16%) and 15.2 million people deceased due to ischemic heart disease and stroke (27%) [1].Thus, improvements in medicine and technology are essential in future gains in longevity and quality of life at the oldest ages. Considering the demographic characteristics of patients, the incidence of CAD increases with age. Men show a higher prevalence and are also more likely to receive coronary interventions as women. CAD is perceived as a male-dominated disease; however, it is the leading cause of death in women aged 65 years and older worldwide. In this context, it is important to consider that ischemic heart diseases in women often progress asymptomatically, which results in delays of medical treatment [2].

In the last two decades percutaneous coronary interventions (PCI) with stent implantation emerged as alternative to surgical procedure of coronary artery bypass grafting (CABG) in the treatment of CAD. This trend is particularly evident in Germany: in 2003 the ALKK (“Arbeitsgemeinschaft leitender kardiologischer Krankenhausärzte”) registry reported approximately 30,000 cases of PCI with stent [3]. However, in 2015 more than 300,000 PCI’s compared to 50,000 procedures of CABG proceeded whereby drug-eluting stents (DES) accounted for 91.2% of revascularizations [4]. Recent findings of comparing the efficacy and safety of coronary stents preferred innovative DES over traditional bare-metal stents (BMS) in terms of a lower incidence of adverse cardiac events leading to death [5]. Thus, we hypothesized survival benefits of DES towards BMS within the first three years since patients underwent initial coronary intervention (Hypothesis I). According to short-term follow-ups of patients with coronary interventions, stent implantation potentially revealed lower mortality rates compared to CABG (Hypothesis II) [6].However, considering the mortality selection shortly after revascularization procedure, we expected CABG to be associated with a higher 3-year-survival compared to DES (Hypothesis III).

The role and benefits of CABG in the treatment of a specific subtype of CAD, namely complex multivessel disease, were well established in the recent past by long term clinical follow up and landmark studies such as the SYNTAX trial. The outcome following PCI or CABG in patients exhibiting left main stem disease was addressed by several trials such as the EXEL trial and the NOBLE trial. These trials reported non-inferiority of PCI in the treatment of left main stem disease but with persistent advantages for CABG in terms of the need for repeated revascularization during follow-up [7, 8].

Contrary to previous studies, our sample was not biased by health selection of respondents. We compared survival after stent- and bypass procedure to mortality in CAD patients who never underwent coronary intervention and also provided the disease duration of CAD. Thus, we represented a valid ‘real-world analysis’ of clinical results following different types of coronary revascularization in a large patient cohort.

Our study complied with RECORD reporting guidelines, which were developed for studies involving routinely collected health data. As an extension of the existing STROBE guidelines, we fulfilled the RECORD reporting requirements by adequately conveying the methods and results of our research.

## Materials and methods

### Data source

Our analysis based on anonymized routine data from the largest public health insurance ‘Allgemeine Ortskrankenkassen’ (AOK) in Germany. The scientific institute of the AOK (WIdO) granted the data access. The random sample consisted of 250,000 members from private households and nursing homes which aged 50+ years, and we tracked from first quarter in 2004 until fourth quarter in 2015 at the latest. The routine data covered patients’ sex, date of birth and death, as well as in- and outpatient diagnoses coded by the Operational and Procedural Classification (OPS), and the International Classification of Diseases and Related Health Problems (ICD-10-GM). The data also comprised a unique identification number and only involved completely anonymized information. Thus, the study complied with the tenets of the Declaration of Helsinki and did not require an ethical approval.

### Definition of outcome measure and risk factors

The mortality status based on master data of health claims. In analysis, we set the time of death to the middle of the month of death.

We included BMS coded by OPS as ‘8–837.k’ and DES coded as ‘8–837.m’. For CABG we considered OPS-codes ‘5–361*’ and ‘5–362*’. We categorized the variable of intervention type into “BMS”, “DES”, “CABG”, “mixed”, and “none” (no procedure). The categories BMS, DES and CABG implied initial interventions, whereas patients in the mixed-group underwent at least two different revascularization techniques in one quarter or received different intervention types as a re-intervention. Patients remained in ‘none’ as long as they did not receive BMS, DES, or CABG. Intergroup changes from none to BMS, DES, CABG or mixed and from BMS, DES or CABG to mixed were possible.

### Control variables and validation strategy

Analysis controlled for sex, and age at first valid CAD diagnosis or at first coronary intervention (50–54, 55–59, 60–64, 65–69, 70–74, 75–79, 80–84, 85+). To identify modifying effects of other cardiac diseases on mortality, we included the following ICD-10-diagnoses ever received between 2004 and 2015: acute coronary syndrome (I21 and I20.0), congestive heart failure (I09.9, I11.0, I13.0, I13.2, I25.5, I42.0, I42.5-I42.9, I43, I50), cardiogenic shock (R57.0), arrhythmia (I44.1-I44.3, I45.6, I45.9, I46, I47, I48, I49, R00.0, R00.1, R00.8, T82.1, Z45.0, Z95.0), multivessel coronary artery disease (I25.13).

We also considered non-cardiovascular diseases with high impact on mortality. We measured the risk factor “multimorbidity” by an additive score as the number of the following acute and chronic diseases ever diagnosed between 2004 and 2015: atherosclerosis (I70), Alzheimer’s disease (F00), diabetes mellitus (I10–I14), cancer (C00–C97), liver diseases (B18, K70–K72, K76, Z94.4), lung diseases (J44, J47), neurological diseases (G00–G09, G23, G24–G26, G31.8, G32), kidney diseases (N17–N19, Z94.2, T82.4, Z99.2), Parkinson’s disease (G20–G22), cerebrovascular diseases (I60–I69, G45, G46, H34), paralysis (G80–G83, G04.1, G11.4) and peptic ulcer (K25–K28). The multimorbidity score consisted three categories: 0–1, 2–6, or 7+ of the selected diseases.

In addition, we considered external injuries (S00–S03, T00, T01.3, T02.4, T02.6–T02.9, T05.8, T05.9, T14–T98) as non-degenerative risk factor of death. All diseases were defined as irreversible since the first observed diagnosis.

Another control variable concerned the duration since coronary intervention measured in years. We compared CAD patients in 1st to 2nd year since intervention with a third (residual) group that covered both, CAD patients in the 3rd year and CAD patients who never received BMS, DES, or CABG.

We reduced the problem of false-positive CAD diagnoses by applying a validation strategy: to define the first CAD diagnosis as valid, the patient required the co-occurrence of CAD diagnosis in another quarter over the whole observation period [9]. We considered all covariates, with the exception of sex and age at incident CAD diagnosis, to be time-varying variables with value 1 since first valid diagnosis and 0 otherwise.

### Sample selection

**Fig. 1:**
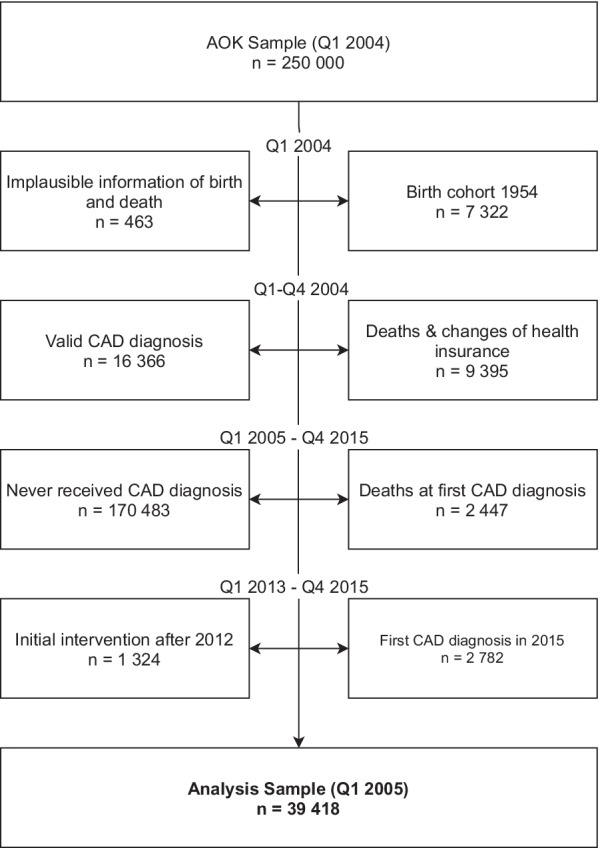
Flowchart of sample selection procedure

First, we excluded all patients with inconsistent or implausible information of birth or death (n = 463) and 7332 persons under the age of 50 (Fig. 1). To only include incident CAD cases, patients with CAD diagnosis already underlying in 2004 (n = 16,366) met the exclusion criteria. Considering deceased and patients who left the AOK in 2004, the sample decreased by 9395 persons. We further excluded patients with first CAD diagnosis in 2015 (n = 2782) to avoid an end-of-study-bias, and those who had less than three years of follow-up after initial intervention as of Q1 2013 (n = 1324), just as patients with an incident CAD diagnosis and death in the same quarter (n = 2447). The final study cohort comprised 39,418 incident CAD patients without or with coronary intervention between Q1 2005 and Q4 2012.

### Statistical analysis

We estimated sex and age-standardized 3-year mortality rates from 2005 to 2015 by using the German average population of 2005 and computed the mortality rate for each intervention type by dividing the number of deaths ($D_{2005 - 15,x,a}$) by the population under risk (Eq. 1). The Person-years under risk ($PY_{Risk}$) from 2005 to 2015 defined the population of risk in the denominator.

1 $$Mortality_{2005 - 15,x,a} = \frac{{D_{2005 - 15,x,a} }}{{PY_{Risk2005 - 15,x,a} }}*100$$

To compare 3-year survival after coronary intervention, we applied Kaplan–Meier estimators. We also computed Cox proportional-hazard models, which aimed to examine disparities in the 3-year mortality among CAD patients with adjustment for demographic characteristics, cardiovascular and non-cardiac diseases and the years passed since intervention. In sensitivity analysis, we calculated an additional model excluding CAD patients who never underwent coronary intervention in the observation period, as well as the observation time prior to intervention. All analyses were conducted with the use of Stata (version 16.1).

We measured analysis time as the years since first valid CAD diagnosis as of Q1 2005 at the earliest (Additional file 1: Fig. S1, persons 1–5). Following the principles of Hernán et al. (2016) and Emilson et al. (2018), we emulated a target trial [10, 11]. We maximally tripled all eligible observations (measured in person-times) and assigned each copy to the corresponding coronary intervention group (none, BMS, DES, CABG or mixed). In case of a group change we defined a time zero of analysis time (Additional file 1: Fig. S1, persons 1 & 2). Coronary intervention simultaneous with the first valid CAD diagnosis (study entry) was also possible (Additional file 1: Fig. S1, persons 3 & 5). To face the immortal time bias, we censored CAD patients after a follow-up of 3 years spend in the category of BMS, DES or CABG (Additional file 1: Fig. S1, persons 2, 3 & 5). Person-times spend in the categories of none and mixed had no observational time limit (Additional file 1: Fig. S1, person 4).

## Results

### Patients at CAD diagnosis and death

12,974 out of 39,418 incident CAD patients died (Table 1) and we found that 1245 of the deceased underwent coronary revascularization (Tab 2). 48.22% of patients with coronary intervention received a BMS, whereas 45.94% of the deceased were assigned to the same group. 22.64% of CAD patients received DES and 18.77% underwent bypass surgery. Mortality in patients with CABG (18.80%) was slightly higher than in patients with DES (17.35%). A total of 754 (10.38%) persons had mixed intervention types. The mixed group consisted of 371 (49.20%) initial revascularizations and 383 (50.80%) re-interventions with changed intervention type.

**Table 1:** Descriptive overview of CAD patients at study entry and death, 2005–2015

	Persons at CAD diagnosis		Deaths 2005–2015	
	n	%	n	%
**Sex**				
Males	19,760	50.13	6149	47.39
Females	19,658	49.87	6825	52.61
**Age at CAD diagnosis/coronary intervention**				
50–54	501	1.27	63	0.49
55–59	2366	6.00	277	2.14
60–64	4144	10.51	556	4.29
65–69	6051	15.35	1175	9.06
70–74	8175	20.74	2032	15.66
75–79	7664	19.44	2693	20.76
80–84	5969	15.14	2951	22.75
85+	4548	11.54	3227	24.87
**Acute coronary syndrome**				
No	28,389	72.02	8366	64.48
Yes	11,029	27.98	4608	35.52
**Congestive heart failure**				
No	21,240	53.88	2359	18.18
Yes	18,178	46.12	10,615	81.82
**Cardiogenic shock**				
No	38,541	97.78	11,515	88.75
Yes	877	2.22	1459	11.25
Arrhythmia				
No	18,775	47.63	3183	25.53
Yes	20,643	52.37	9791	75.47
**Multivessel coronary artery disease**				
No	28,106	71.30	8593	66.23
Yes	11,312	28.70	4381	33.77
**Comorbidities**				
0–1	3283	8.33	500	3.85
2–6	30,076	76.30	9927	76.51
7–12	6059	15.37	2547	19.63
**External injuries**				
No	30,610	77.65	9343	72.01
Yes	8808	22.35	3631	27.99
**Years since intervention**				
First year	6880	28.70	518	3.99
Second year	–	–	354	2.73
Third year/no intervention	32,538	71.30	12,102	93.28
Total	39,418	100%	12,974	100%

**Table 2:** Coronary interventions 2005–2012 and deaths 2005–2015

	Coronary interventions 2005–2012		Deaths 2005–2015	
	n	%	n	%
BMS	3502	48.22	572	45.94
DES	1644	22.64	216	17.35
CABG	1363	18.77	234	18.80
Mixed	754^a^	10.38	223	17.91
Total	7263	100%	1245	100%

^a^Including 371 (49.20%) initial coronary interventions and 383 (50.80%) re-interventions with changed intervention type

The sample revealed a balanced representation of females and males at first CAD diagnosis (50.13% and 49.87%) and among the deceased (47.39% and 52.61%, Table 1). With increasing age, the number of CAD diagnoses increased as well as mortality. 20.74% received their first CAD diagnosis in the age of 70–74, whereas mortality reached a peak at ages 85 and older (24.87%). Considering the other ischemic heart diseases at study entry, acute coronary syndrome was diagnosed in 27.98% and almost half of the insured (46.12%) had congestive heart failure. Cardiogenic shock affected 2.22% of patients and we found a prevalence of 52.37% for arrhythmia. 28.70% suffered from multivessel coronary artery disease at first CAD diagnosis. Among the deceased, cardiogenic shock showed a five times higher prevalence (11.25%) compared to study entry and patients with congestive heart failure contributed the majority (81.82%). We found arrhythmia in 75.47% whereas diagnoses of acute coronary syndrome (35.37%) and multivessel coronary artery disease (33.77%) concerned approximately one third of deaths.

Most patients had at least two to six additional diseases independent from cardiac diseases at study entry (76.30%) and death (76.51%). 22.35% were affected by external injuries at first valid CAD diagnosis and 27.99% of patients at death. 6880 (28.70%) had initial revascularization at first CAD diagnosis. Among the deceased, 93.28% never received coronary intervention, or intervention was more than three years ago. 2.73% of deaths occurred in the second year since intervention whereas 3.99% of patients died in the first year.

The descriptive overview of coronary interventions per patient (2005–2012) and subsequent deaths (2005–2015) contained initial interventions as well as re-interventions with same and changed intervention type (Additional file 2: Table S1). 78.44% received one intervention. 16.98% underwent a second intervention whereas 3.62% obtained a third re-vascularization. The number of coronary interventions per person varied between one and seven. CAD patients with four and more interventions represented less than 1% of the sample.

### Survival in CAD patients

**Fig. 2:**
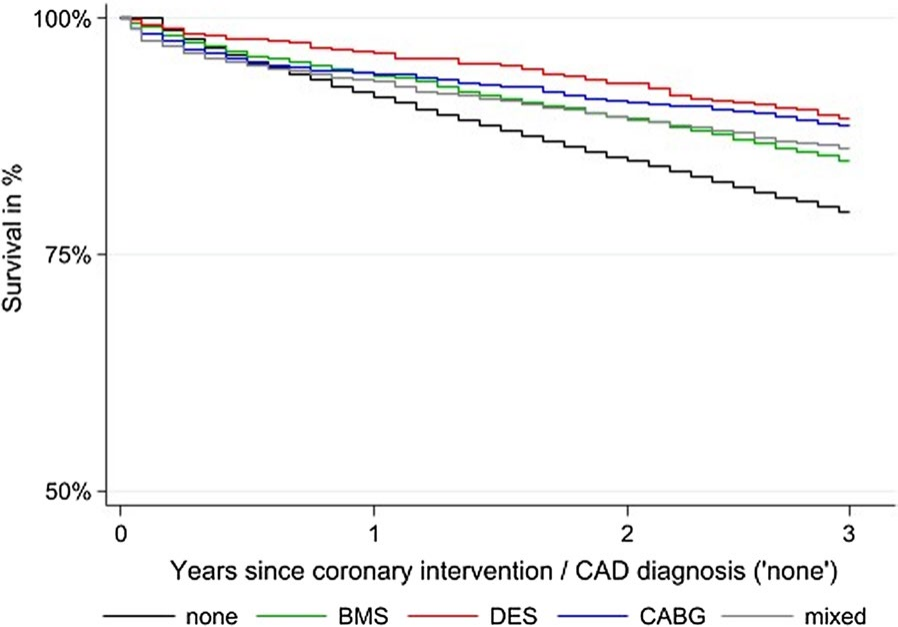
Kaplan–Meier survival curves by coronary intervention group

Without risk adjustment, the survival curves of BMS, CABG, mixed and none overlapped within the first year since coronary intervention (Fig. 2, Additional file 2: Table S2), whereas after one year, DES patients showed a higher survival (DES: 96.31%) compared to BMS (93.99%); CABG (94.12%) or the none group (91.65%). With progressing time, the survival curves revealed disparities in mortality risk. Whereas CABG implied higher mortality compared to BMS, DES and none after the first year since intervention, CABG had survival advantage over BMS and none (CABG: 91.19%; BMS: 89.42%; none: 84.87%) after the second year as well as after the third year (CABG: 88.71%; BMS: 84.99%; none: 79.54) and converged to the curve of DES. We found worse survival outcomes for CAD patients without coronary intervention. Three years after CAD diagnosis just over two thirds of the none group were still alive. The Log-rank test indicated non-equality of survivor functions (*p* < 0.001).

Within the first three years after intervention, mortality rates increased with increasing age of patient but there was no sex difference by intervention type (Additional file 3: Fig. S2). Mortality rates of CAD patients without revascularization also showed no significant sex disparities.

### Multivariable analysis

Controlled for sex, age at first CAD diagnosis or coronary intervention, cardiovascular and non-cardiac diseases as well as the years since intervention, mortality in patients without stent or bypass surgery was 60% (*p* < 0.001) higher compared to any intervention type (Table 3). DES patients showed 21% (*p* = 0.004) lower mortality risk as the BMS group. We also observed survival benefits of CABG (HR 0.79; CI 0.68–0.92; *p* = 0.002) towards BMS patients. In sensitivity analysis (Additional file 3: Table S3), the HR for DES (HR 0.77; CI 0.66–0.90; *p* = 0.001) and CABG (HR 0.68; CI 0.58–0.80; *p* < 0.001) even decreased as compared to the total model, indicating that the effects of coronary interventions were not driven by mortality pattern of the CAD patients without BMS, DES, or CABG.

**Table 3:** Multivariable analysis of risk factors effecting mortality in CAD patients, 2005–2015

	Hazard Ratio	(95% CI)	*p*-value
Coronary intervention (Reference: BMS)			
None	1.60	(1.41–1.82)	*p* < 0.001
DES	0.79	(0.68–0.93)	0.004
CABG	0.79	(0.68–0.92)	0.002
Mixed	0.81	(0.69–0.94)	0.007

(n = 39,418; deaths = 12,974; LR = 12,032.08; *p* < 0.001)

Cox model controlled for sex, age at CAD diagnosis/coronary intervention, cardiovascular and non-cardiac diseases, years since intervention

## Discussion

DES and CABG showed similar survival advantages in 3-year mortality follow-up which based on a large number of CAD patients. Among the different operative procedures, BMS indicated worse results but worst survival outcomes we found for CAD patients who never underwent coronary intervention. The descriptive results also confirmed a more frequently application of stents compared to bypass surgery. Thus, we are able to replicate the findings of earlier studies but extended them to a longer follow-up period and used a larger sample size with higher representativity of the general population.

When interpreting the results of our study it is important to consider that the analysis included various types of CAD morphology. Earlier studies showed disparities in survival outcomes between DES and CABG for specific CAD profiles. With the adjustment for risk factors of death in multivariable analysis, the tendency of DES to be superior over CABG in Kaplan–Meier estimates ceased. The survival curves implied a higher postoperative mortality risk of open-surgical procedure in first year after intervention compared to catheter-based revascularization, but also showed the long-term benefit of CABG after 3 years of follow-up. Our study confirmed non-inferiority for DES as compared to CABG and thus indicated favorable results for aging populations with high burden of ischemic heart disease. However, the equality of survival outcomes for DES and CABG possibly emerged due to the healthier and younger CAD patients who received bypass surgery. On the other hand, it can be estimated that less complex CAD, i.e. one- and two vessel disease, was preferentially treated by DES regardless patients age. High mortality in patients who never received coronary intervention can be caused by a delayed first diagnosis of CAD, a general worse health profile or patient’s preferences to reject an operative procedure.

Furthermore, our study revealed less expected results. Patients with BMS showed worse survival outcomes as compared to DES or CABG. Nevertheless, BMS represents the most frequent procedure used between 2005 and 2012. According to the current state of medical research, the BMS can be seen as historical in clinical importance. Hence, there is seldom a compelling reason to prefer BMS to DES [12]. Drug-eluting stents were developed to improve the long-term efficacy and safety of patients receiving percutaneous coronary interventions and hitherto represents the gold standard in interventional CAD treatment [13].

Given the generally higher number of deaths in male CAD patients, the missing sex-gradient in mortality in our study supports earlier studies that point towards a more severe progression of disease in women due to a delayed first CAD diagnosis.

Prospective clinical trials demonstrated clinical advantages regarding survival and/or subsequent cardiac events for either treatment method depending on specific CAD morphologies, e.g. isolated left main stenosis or three-vessel disease [14]. A large retrospective study by Head et al. (2018) analyzed differences in mortality following PCI or CABG in patients with multivessel-disease and left main stem stenosis. Similar to the mentioned prospective clinical trials this pooled analysis of individual patient data found a mortality benefit for CABG over PCI in patients with multivessel-disease whereas no benefit for CABG over PCI was seen in left main disease [15]. The strength and main clinical impact of the retrospective results presented here is that data were obtained from a large patient cohort without pre-specifying for certain coronary morphologies including even one- and two-vessel disease. In this way our data provided a unique validation of early and midterm survival following invasive CAD treatment on an observational basis in “a real-world scenario and matched the overall survival results following PCI or CABG performed within prospective clinical trials.

### Strengths and limitations

An important advantage of this health claims analysis was the large sample size concerning the number of CAD patients and different coronary interventions, just as the range of diagnoses from all fields of medicine**.** Also, the cohort design offered a long observation period of twelve years to track patient’s health status and changes over time. We reduced false-positive diagnoses of CAD by using a validation strategy and assumed high validity of the diagnoses, which based on medical examinations of practicing physicians, and the interventions because health claims data aims to document medical treatments and their costs.

The elimination of false-negative CAD diagnoses was possible due to the wash-out period in 2004 but came up again when patients received their second diagnosis of coronary artery disease after 2014. Another limitation concerned that our data based on all-cause mortality and was limited to medical or demographic risk factors of death. Also, the patient’s preferences have to be considered, who often favor a less invasive treatment strategy.

In contrast to clinical trials, our official process-generated data source (‘AOK’) was not affected by self-selected dropouts, reactivity (e.g. social desirability), any response biases and any cognitive biases (e.g. recall bias or overconfidence bias). To solve three general problems of observational studies: The mortality selection bias, the end-of-study-bias, and the immortal time bias, we opposed the biases by emulating a randomized target trial following pre-defined eligibility criteria [10].

## Conclusion

Our results strengthened the interdisciplinary approach in treating CAD patients by either percutaneous coronary intervention using DES or CABG are clearly demonstrated to represent complementary rather than competing treatment options. An early survival benefit of PCI using DES is supplemented by an advantage of CABG regarding the mid and long-term survival. Similar to the treatment of structural heart disease, institutional Heart Teams consisting of cardiac surgeons and cardiologists should evaluate CAD cases on a routine basis and allocate patients to the optimal treatment, thereby taking in account patient age, comorbidities and the specific morphology (i.e. single or multi-vessel disease, presence or absence of left main stenosis) of the underlying CAD. There is a need for future long-term observational analyses, such as the one presented here, but with a focus on specific coronary morphologies in order to compare their results of surgical versus interventional CAD treatments in large cohorts of patients.

## Supplementary Information

**Additional file 1:** Scheme of included and excluded person-times in the study using the example of five fictitious persons with different study entries and exits and health histories.

**Additional file 2: Table S1.** Number of coronary interventions per patient 2005–2012 and deaths 2005–2015. **Table S2.** Kaplan-Meier survival estimates by coronary intervention, survival in % after 1, 2 and 3 years, 2005–2015. **Table S3.** Sensitivity analysis: multivariable analysis: Cox model adjusted for risk factors effecting mortality in CAD patients with coronary interventions, 2005–2015a.

**Additional file 3: Fig. S2.** Age-specific and age-standardized 3-year mortality rates of CAD-patients by sex and coronary interventions from 2005 to 2015: BMS (A), DES (B), CABG (C), mixed (D) or none (E).

## Data Availability

The scientific research institute of the AOK (WIdO) has strict rules regarding data sharing because health claims data are a sensitive data source and have ethical restrictions imposed due to concerns regarding privacy. Anonymized data are available to all interested researchers upon request. Interested individuals or an institution who wish to request access to the health claims data of the AOK may contact the WIdO (webpage: http://www.wido.de/, mail: wido@wido.bv.aok.de).
