# Supplementary material for: Stents versus bypass surgery: 3-year mortality risk of patients with coronary interventions aged 50+ in Germany
Source: J Cardiothorac Surg. 2022 Oct 1;17:246. doi: 10.1186/s13019-022-02014-2 (PMC9526318; doi:10.1186/s13019-022-02014-2)
Supplement: Supplementary file 1 — Additional file 1: Scheme of included and excluded person-times in the study using the example of five fictitious persons with different study entries and exits and health histories. [file 13019_2022_2014_MOESM1_ESM.pdf]

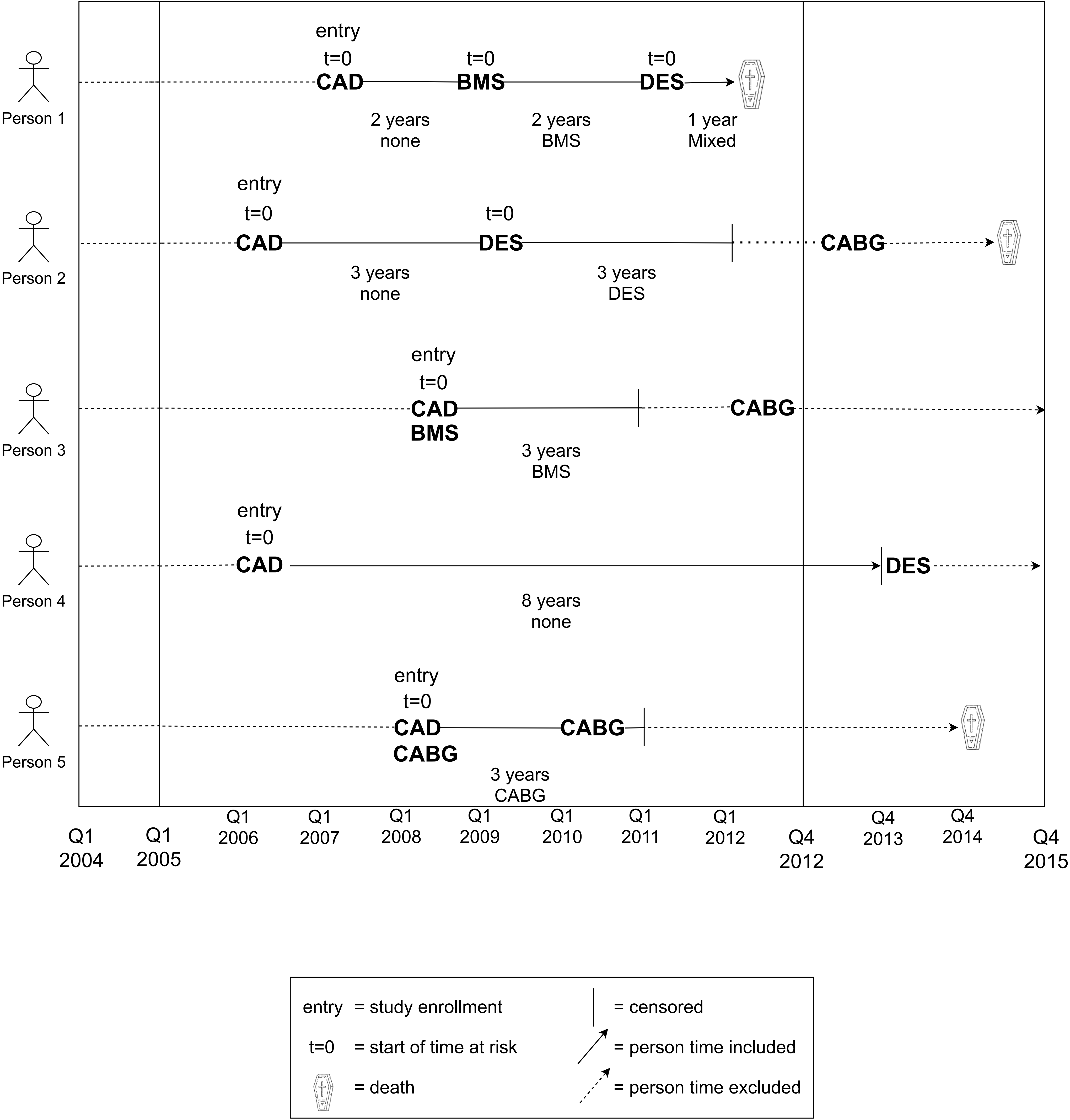
Person 1
entry
t=0
CAD
t=0
BMS
t=0
DES
2 years
none
2 years
BMS
1 year
Mixed
Person 2
entry
t=0
CAD
t=0
DES
CABG
3 years
none
3 years
DES
Person 3
entry
t=0
CAD
BMS
CABG
3 years
BMS
Person 4
entry
t=0
CAD
DES
8 years
none
Person 5
entry
t=0
CAD
CABG
CABG
3 years
CABG
Q1 2004
Q1 2005
Q1 2006
Q1 2007
Q1 2008
Q1 2009
Q1 2010
Q1 2011
Q1 2012
Q4 2012
Q4 2013
Q4 2014
Q4 2015
entry = study enrollment
t=0 = start of time at risk
= death
= censored
= person time included
= person time excluded
