# Supplementary material for: Stents versus bypass surgery: 3-year mortality risk of patients with coronary interventions aged 50+ in Germany
Source: J Cardiothorac Surg. 2022 Oct 1;17:246. doi: 10.1186/s13019-022-02014-2 (PMC9526318; doi:10.1186/s13019-022-02014-2)
Supplement: Supplementary file 2 — Additional file 2: Table S1. Number of coronary interventions per patient 2005–2012 and deaths 2005–2015. Table S2. Kaplan-Meier survival estimates by coronary intervention, survival in % after 1, 2 and 3 years, 2005–2015. Table S3. Sensitivity analysis: multivariable analysis: Cox model adjusted for risk factors effecting mortality in CAD patients with coronary interventions, 2005–2015a. [file 13019_2022_2014_MOESM2_ESM.docx]

**Supplementary material**

Table S1. Number of coronary interventions per patient 2005-2012 and deaths 2005-2015.

|  |  | **Coronary interventions per patient 2005-2012** | | **Deaths 2005-2015** | |
| --- | --- | --- | --- | --- | --- |
|  |  |  |  |  |  |
|  |  | **n** | **%** | **n** | **%** |
| Number of interventions per patient | | |  |  |  |
|  | 1 | 5 397 | 78.44 | 993 | 79.76 |
|  | 2 | 1 168 | 16.98 | 198 | 15.90 |
|  | 3 | 249 | 3.62 | 42 | 3.37 |
|  | 4 | 59 | 0.86 | 10 | 0.80 |
|  | 5 | 5 | 0.07 | 2 | 0.16 |
|  | 6 | 1 | 0.01 | 0 | 0.00 |
|  | 7 | 1 | 0.01 | 0 | 0.00 |
| **Total** | | **6 880** | **100%** | **1 245** | **100%** |

Table S2. Kaplan-Meier survival estimates by coronary intervention, survival in % after 1, 2 and 3 years, 2005-2015.

|  | **Years since coronary intervention / CAD-diagnosis ('None')** | | | | | |
| --- | --- | --- | --- | --- | --- | --- |
|  | **1** | | **2** | | **3** | |
|  | **%** | **95%-CI** | **%** | **95%-CI** | **%** | **95%-CI** |
| **none** | 91.65% | (91.33%-91.95%) | 84.87% | (84.46%-85.26%) | 79.54% | (79.07%-80.00%) |
| **BMS** | 93.99% | (93.21%-94.69%) | 89.42% | (88.41%-90.35%) | 84.99% | (83.91%-86.08%) |
| **DES** | 96.31% | (95.40%-97.03%) | 93.09% | (91.91%-94.11%) | 89.37% | (87.94%-90.63%) |
| **CABG** | 94.12% | (93.03%-95.05%) | 91.19% | (89.89%-92.33%) | 88.71% | (87.27%-90.00%) |
| **mixed** | 93.30% | (91.99%-94.41%) | 89.28% | (87.65%-90.70%) | 86.25% | (84.42%-87.88%) |

Table S3. Sensitivity analysis: multivariable analysis: Cox model adjusted for risk factors effecting mortality in CAD patients with coronary interventions, 2005-2015^a^.

|  |  | **Hazard Ratio** | **(95% CI)** | | | | | **p-value** |
| --- | --- | --- | --- | --- | --- | --- | --- | --- |
| Coronary intervention (Reference: BMS) | | |  |  |  |  |  |  |
|  | DES | 0.77 | ( | 0.66 | - | 0.90 | ) | 0.001 |
|  | CABG | 0.68 | ( | 0.58 | - | 0.80 | ) | p<0.001 |
|  | mixed | 0.76 | ( | 0.64 | - | 0.89 | ) | 0.001 |
| (n=6 880; deaths=1 245; LR=1 953.01; p<0.001) | | | | | | | | |
| ^a^Cox model controlled for sex, age at CAD diagnosis / coronary intervention, cardiovascular and non-cardiac diseases, years since intervention | | | | | | | | |
|  |  |  |  |  |  |  |  |  |
