# Supplementary material for: Stents versus bypass surgery: 3-year mortality risk of patients with coronary interventions aged 50+ in Germany
Source: J Cardiothorac Surg. 2022 Oct 1;17:246. doi: 10.1186/s13019-022-02014-2 (PMC9526318; doi:10.1186/s13019-022-02014-2)

Figure 4. Age-specific and age-standardized 3-year mortality rates of CAD-patients by sex and coronary interventions from 2005 to 2015: BMS (A), DES (B), CABG (C), mixed (D) or none (E)

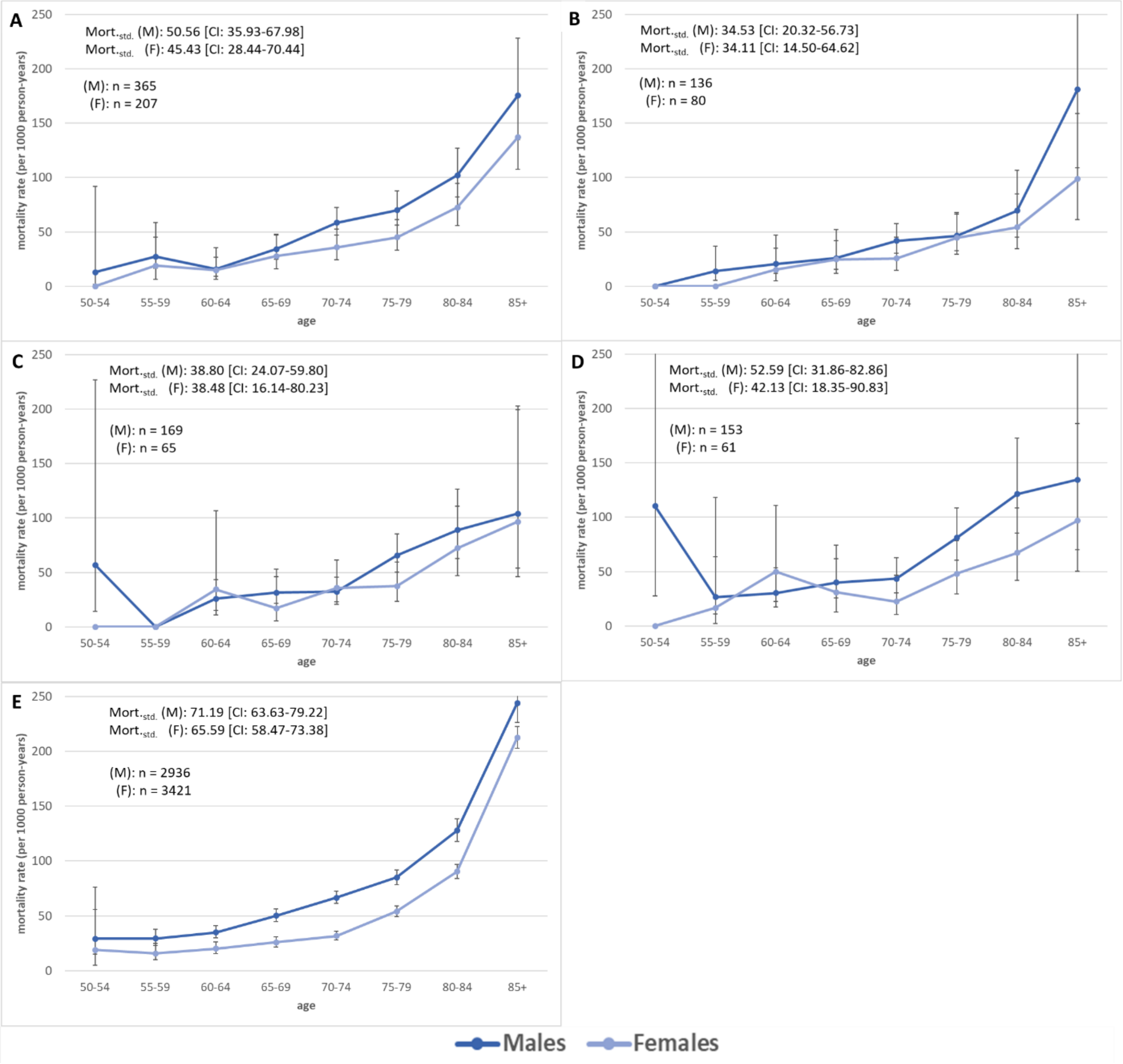

Supplement: Supplementary file 3 — Additional file 3: Fig. S2. Age-specific and age-standardized 3-year mortality rates of CAD-patients by sex and coronary interventions from 2005 to 2015: BMS (A), DES (B), CABG (C), mixed (D) or none (E). [file 13019_2022_2014_MOESM3_ESM.pdf]
